# CSF biomarkers of immune activation and Alzheimer's disease for predicting cognitive impairment risk in the elderly

**DOI:** 10.1002/alz.093743

**Published:** 2025-01-09

**Authors:** Francis Shue, Launia J White, Rachel Hendrix, Jason D Ulrich, Rachel L. Henson, William C. Knight, Yuka A Martens, Ni Wang, Bhaskar Roy, Skylar C Starling, Yingxue Ren, Chengjie Xiong, Yan W. Asmann, Jeremy A. Syrjanen, Maria Vassilaki, Michelle M. Mielke, Carlos Cruchaga, David M. Holtzman, Guojun Bu, Ronald C. Petersen, Michael G. Heckman, Takahisa Kanekiyo

**Affiliations:** ^1^ Mayo Clinic, Jacksonville, FL USA; ^2^ Washington University in St. Louis, St. Louis, MO USA; ^3^ Hope Center for Neurological Disorders, Washington University School of Medicine, St. Louis, MO USA; ^4^ Knight Alzheimer Disease Research Center, Saint Louis, MO USA; ^5^ Washington University School of Medicine, St. Louis, MO USA; ^6^ The Charles F. and Joanne Knight Alzheimer Disease Research Center, St. Louis, MO USA; ^7^ Mayo Clinic, Rochester, MN USA; ^8^ Wake Forest University School of Medicine, Winston‐Salem, NC USA; ^9^ Washington University School of Medicine, Saint Louis, MO USA; ^10^ Department of Neurology, Mayo Clinic, Rochester, MN USA

## Abstract

**Background:**

The immune system is substantially involved in the development and progression of age‐related cognitive decline and Alzheimer’s disease (AD).

**Method:**

As genetic and environmental factors interactively impact these conditions, we investigated how risk factors such as APOE genotype, age, and sex influence immune activation markers and AD biomarkers in cerebrospinal fluid (CSF) in elderly individuals enrolled in the Mayo Clinic Study of Aging cohort. Among cognitively unimpaired individuals aged over 65 at the baseline visit (N=298), we measured 365 CSF immune activation markers using the proximity extension assay.

**Result:**

We found that age, sex, and diabetes status are associated with altered CSF levels of immune activation markers independently of other factors. For CSF AD biomarkers, we observed significant positive correlations between age and total tau, phosphorylated tau‐181 (p‐tau181), neurofilament light (NfL), and YKL40. APOE4 was also associated with lower Aß42 and higher SNAP25 in CSF. We further examined whether baseline visit variables can predict cognitive decline, represented by the conversion from CDR=0 to CDR>0. We found that age, Aß42, NfL, and REG4 were independently correlated with CDR conversion risk. When the cohort was dichotomized by their median values, older participants with lower Aß42, higher NfL, and higher REG4 at baseline developed cognitive impairment during the follow up with a c‐index of 0.762 while age alone had a c‐index of 0.699.

**Conclusion:**

Together, our results suggest that assessing CSF immune activation markers and AD biomarkers can improve the prediction of cognitive impairment risk in the elderly.

